# Co-Dependency of IAQ in Functionally Different Zones of Open-Kitchen Restaurants Based on Sensor Measurements Explored via Mutual Information Analysis

**DOI:** 10.3390/s23177630

**Published:** 2023-09-03

**Authors:** Monika Maciejewska, Andi Azizah, Andrzej Szczurek

**Affiliations:** Faculty of Environmental Engineering, Wroclaw University of Science and Technology, Wybrzeże Wyspiańskiego 27, 50-370 Wroclaw, Poland; andi.azizah@pwr.edu.pl (A.A.); andrzej.szczurek@pwr.edu.pl (A.S.)

**Keywords:** indoor air quality, open space, VOCs, sensor, restaurant, mutual information

## Abstract

High-quality indoor air is essential in open-kitchen restaurants for ensuring a healthy workplace and comfortable conditions for visitors. In this study, indoor air quality interdependence between the kitchen and the dining zones in open-kitchen restaurants was analyzed. The method was based on measurements of selected air parameters using a sensor technique and mutual information (MI) analysis. A long-term approach (based on a several-hour time series) and a short-term approach (based on a several-minute time series) were applied. This study involved four open-kitchen restaurants. The indoor conditions were represented by the temperature, relative humidity, CO_2_ concentration, and content of the total volatile organic compounds (TVOC) in the air. The MI analyses showed that the long-term co-dependence of the indoor conditions between the kitchen and the dining zones was smaller during business hours (MI = 0.12 ÷ 0.40) compared to night hours (MI = 0.24 ÷ 0.58). The ranking of the long-term MI values for the individual air parameters was MI_CO2_ (0.34) ≅ MI_T_ (0.34) > MI_RH_ (0.28) > MI_TVOC_ (0.23)_._ The short-term interdependencies were smaller during night hours (median MI = 0.01 ÷ 0.56) compared to business hours (MI = 0.23 ÷ 0.61). Additionally, the short-term MI was subject to high temporal variability. The ranking of the short-term MI values for the individual air parameters was MI_CO2_ (0.48) > MI_T_ (0.46) > MI_RH_ (0.37) > MI_TVOC_ (0.26). Due to the weak and highly variable co-dependence of the air parameters between the kitchen and dining areas, multi-zone monitoring of air parameters with an emphasis on TVOC measurements is recommended to ensure proper indoor conditions in open-kitchen restaurants. The presented approach may be applied to design indoor air quality monitoring and ventilation systems not only in open-kitchen restaurants but also in other interiors with functionally different zones.

## 1. Introduction

Openness is an attractive conceptual paradigm in modern design. It has been accommodated in a variety of settings like offices, retail objects, home interiors, restaurants, coffee shops, etc. Technically, an “open-concept” design refers to building interiors with fewer walls and doors. However, it should be underlined that this approach has broad implications for social relations, productivity, and privacy [1]. 

Open-kitchen restaurants became popular in the 1990s [2]. This concept has become one of the biggest trends in the restaurant business in recent years. The open-kitchen design offers many opportunities [3,4], e.g., transparency. Customers can see exactly who is cooking their food, how their meal is prepared, and under what conditions as well as directly enjoy the skills of the chef. It was found that clients of open-kitchen restaurants are more forgiving toward service failures because this design enables them to easily collect direct and circumstantial information about the cause of failure [5]. Open-kitchen restaurants provide a multi-sensory experience that stimulates the appetite of diners. At the same time, their customers are relatively generous about cooking smell service failures [2]. Open-kitchen restaurants build up better interaction with customers who appreciate the artistry of cooking [6]. This fact induces changes in kitchen chefs’ education. The training currently focused on traditional cooking skills needs to be complemented with customer service as a necessary component [7]. In addition, this popular design option allows for saving a lot of construction areas that can be equipped with various appliances for decorating the premises and impressing clients [3,4]. 

To be precise, open-kitchen restaurants offer not only opportunities. They also generate significant problems that can hamper the business. One of these problems is associated with indoor air quality (IAQ) [8] Good IAQ is essential for ensuring a healthy workplace for restaurant personnel and comfortable conditions for visiting customers. The study by Chang [9] revealed that working or frequently dining in an open-kitchen restaurant where grilling or frying takes place caused exposure to harmful levels of PM10 and PM2.5, which were likely to cause respiratory health problems. 

There are two critical zones in open-kitchen restaurants: the kitchen and dining zone. The emission of air pollutants takes place mainly in the kitchen. The cooking processes and other restaurant activities release various air contaminants directly into the indoor environment. These air contaminants include particulate matter (PM) fractions PM2.5 and PM10 [9,10,11,12], carbon-containing compounds, including VOCs [13,14], as well as inorganic compounds, e.g., CO [11] and NO_2_ [15]. Most of them can increase the health risk of the exposed persons, resulting in a higher number of sick days and decreased productivity. Hence, the reduction of air pollution in restaurants’ kitchens may bring significant economic benefits. 

The dining zone Is the next most essential space of an open-kitchen restaurant. Due to the lack of separation from the kitchen, air quality in the dining area is affected not only by customers’ bio effluents but primarily by unwanted emissions from the kitchen. Relatively high concentrations of CO_2_, CO, PM10, PM2.5 benzene, toluene, methylene chloride, and chloroform were measured in the dining areas of Korean barbecue style and Chinese hot pot restaurants [11]. Significant exposures to low carbon-containing species (C1–C4), including, formaldehyde, acetaldehyde, and acetone, were found in the dining areas of six different restaurant types in Kaohsiung, southern Taiwan [13]. Customers are mostly unaware of the health risks associated with dining in open-kitchen restaurants. However, discomforts in the dining zone, e.g., caused by food preparation-related smells, oftentimes lead to complaints and bad reviews, which can damage the business’s reputation. Thus, restaurants take care to give their guests not only the expected dining experience but also to ensure appropriate air quality.

Open-kitchen restaurants have specific requirements for indoor air quality. Well-designed, properly regulated ventilation with high amounts of fresh air allows for the provision and maintenance of the safety and comfort of personnel and diners. Open-kitchen restaurants can be ventilated either naturally, with openings such as windows and/or vents, with a mechanical ventilation system, or with a combination of the two. Control over the air quality inside open-kitchen restaurants requires ensuring an appropriate air exchange between the outdoors and gas circulation between the various zones of the premises. Due to the character and limitations of mechanical and natural ventilation as well as the diversity of the physical conditions within premises, this task may be a big challenge.

Open-kitchen restaurants are complex microenvironments that may vary in terms of size, technical infrastructure, and functionalities. In addition, mechanical and natural ventilation generate complex air flows indoors. Their role in reducing exposure has been investigated in a model study [16], but the results have limited generalization potential. It is difficult to assess the contribution of the particular factors to indoor air quality in different parts of these objects in general.

The objective of this work was to examine the interdependence of air quality between the kitchen and dining zones in open-kitchen restaurants. This aim was justified by the fact that management of IAQ in open-kitchen restaurants requires specific information about the mutual influence of the air between zones in these premises. This paper aims to show that sensor technology and dedicated data analysis allow us to attain such information. Mutual information (MI) analysis of the air parameters was proposed to provide a probabilistic evaluation of their co-dependence in the two zones based on the indoor air monitoring data.

MI analysis is effective at detecting versatile associations in the data [17] and contributes to the understanding of the mechanisms behind data intercorrelations [18,19]. The results of the MI analysis agree with what is known about the physics of the phenomena represented by the data [20]. One of the domains of successful MI application in environmental engineering is the feature selection aimed at choosing the predictors for air quality models [21,22,23]. MI analysis effectively supports the design of air quality monitoring networks [24], their evaluation [25], maximizing their performance [26,27], and planning network evolutions [24,25].

To our knowledge, this work is the first attempt to apply MI to the examination of the co-dependence of indoor conditions between functionally different zones in an open space. The examination of this issue plays a principal role in assessing the validity of multi-zone monitoring of indoor air quality. Various monitoring options have become thinkable due to large progress in sensor techniques. Increasing attention has been recently paid to wearable sensor development [28,29]. Wearable sensors open the possibility of determining the personal comfort of occupants upon exposure to versatile indoor conditions.

The rest of this paper is structured as follows: Section 2 provides the details of this experimental study. The open-kitchen restaurants involved in this study are characterized, and a description of the multi-sensor measurement devices is included. Section 3 is dedicated to the methods of data analysis. Firstly, the data pre-processing stage is briefly addressed. Secondly, the general concept of MI is introduced together with the applied computation algorithm. Thirdly, the MI application to explore the co-dependence of the indoor conditions between two zones of an open space is characterized. Section 4 presents the results of the qualitative and quantitative analysis of the co-dependence of indoor conditions in functionally different zones of open-kitchen restaurants. MI between zones was investigated for the selected air parameters in the long-term and short-term perspectives. The problem of the temporal variability of MI was addressed in the context of restaurant operation schedules. The results of our work are discussed in Section 5. The conclusions are presented in Section 6.

## 2. Experimental Section

**Figure 1:**
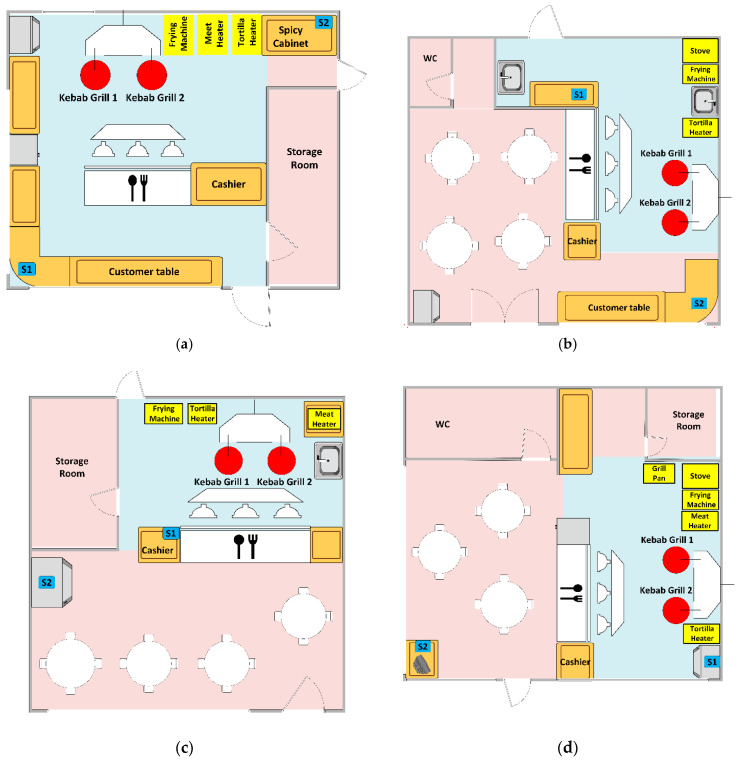
The layout of open-kitchen restaurants involved in this study. (**a**) Object 1; (**b**) Object 2; (**c**) Object 3; (**d**) Object 4.

This measurement study was carried out in four open-kitchen restaurants of kebab-style cuisine. Basic information concerning open-kitchen restaurants involved in this study is included in Table 1. Their layouts were presented in Figure 1. We headed to restaurants offering quick-service and serving a substantial number of city inhabitants. The objects included in this study were representative of this group. They have several common features. Restaurants served food that consisted of similar ingredients (meat, vegetables) and was prepared similarly (mostly grilling and frying). They had a small size, which implies a small dining zone and still smaller kitchen zone that were fully connected. Restaurants were similarly equipped (grills, deep fryer, cooker, fridge, sink, etc.). The customer rotation was high, and the time of consumption was short. The air exchange was realized by natural ventilation, including through open windows and doors. Fumes from cooking and grilling were removed by the manually controlled exhaust hoods located above the grills and frying machine. The uniqueness of individual restaurants was quite limited. It was associated with their location (city center, residential area, etc.), implying slightly different customer segments, number of clients, operating hours, actual size of interior, window and door opening habits, and personnel routines. The number of restaurants involved in this study was limited due to difficulty in attaining consent to perform measurements during regular functioning.

The interior layout of the restaurants IIded in this study is shown schematically in Figure 1. In each facility, the kitchen zone and the restaurant zone could be distinguished. The division of space is conventional since the zones have different functions. There are no physical barriers between them in the form of walls or other dividers. The zones are equipped differently. Different activities take place there, which are realized by different people. In the kitchen zone, meals are prepared. Here, there are grills, a stove, a sink, a refrigerator, and tabletops for cutting ingredients as well as composing and serving dishes. It is accessible only to staff. The dining area is where the consumption of meals takes place. Tables or tabletops are located here. The zone is accessible to the restaurant’s guests and staff. During the restaurant’s operating hours, different conditions are expected in each of the zones that are suitable for the people who are there and the tasks these people perform.

Selected air parameters were monitored in the facilities. Measurements were carried out simultaneously in the kitchen and dining areas using individual sensor devices. The location of measurement points in each facility is shown in Figure 1, and the distances between them are given in Table 1. Measurement sessions took place in summer and lasted from five to seven days in each facility (Table 1).

Multi-sensor devices were used, which enabled the measurement of multiple air parameters using a single device.

**Figure 2:**
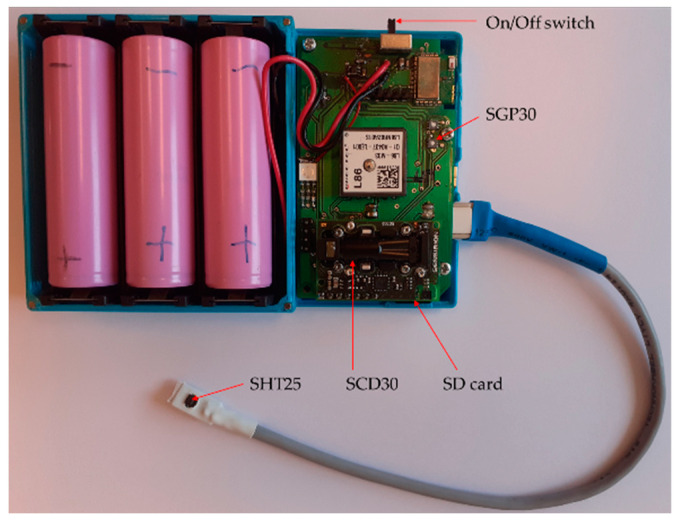
Inside of the multi-sensor device applied in this study.

The multi-sensor devices were developed at Wroclaw University of Science and Technology. The individual device is presented in Figure 2. It is small (12 × 8 × 2.5 cm), self-contained, and automatic. It collects the measurement data with a temporal resolution of 2 s. The data were saved on an SD card. Batteries assure continuous, unattended operation of the device for at least five days. Table 2 quotes the details of the sensors mounted in the device that measured indoor air parameters analyzed in this study. They were temperature, relative humidity, carbon dioxide concentration, and total volatile organic compounds. In the case of SGP30, we utilized raw measurement signals. SGP30 sensor signal increase corresponds to the decreasing content of TVOC in the air.

## 3. Materials and Methods

### 3.1. Data Pre-Processing

Data pre-processing plays a vital role in data analysis by enhancing data quality and preparing it for MI calculations. The process consists of outlier removal, data merging, and interpolation. Outlier removal involves identifying and eliminating data points that fall outside the acceptable range. This process ensures that extreme values do not unduly influence the analysis outcomes. Tukey’s rule was applied to remove the outliers [31]. Firstly, we calculated the first quartile ($Q_{1}$) and third quartile ($Q_{3}$) of each measured air parameter. Then, the interquartile range (IQR) was defined using the formula given by Equation (1). Finally, the lower bound (LB) and upper bound (UB) are given by Equations (2) and (3), respectively. Tukey recommends a predefined constant r = 1.5. Data points either higher than *UB* or located below *LB* are categorized as outliers.

(1) $$IQR=Q_{3}-Q_{1}$$

(2) $$LB=Q_{1}-r\cdot IQR$$

(3) $$UB=Q_{3}+r\cdot IQR$$

The outlier removal process was conducted individually for each restaurant. The data were grouped based on the zone (kitchen and dining area). Then, the outliers were removed for each air parameter one by one.

Another important aspect of data pre-processing is data merging, where data from different sources are combined to create a comprehensive dataset. In this study, a merging technique based on an inner join approach was employed, retaining only the data points with matching date–time values. This procedure ensures that the resulting dataset has consistent data lengths across the two locations. However, it should be noted that this merging process may introduce gaps in the time series data, necessitating further treatment.

To address these gaps, interpolation was performed as the third step of data pre-processing. Interpolation involves estimating missing values by leveraging the values of neighboring data points. The linear method was applied because it is supported for data series with a multi-index. It ignores the index and treats the values as equally spaced. The concept assumes a linear relationship between data points and fills in missing values by estimating them as a linear function of neighboring data points. We determined the missing value (*y*) using the equation of a straight line to connect the two data points as shown in Equation (4) [32].

(4) $$y=y_{1}+\left(x-x_{1}\right)(\frac{y_{2}-y_{1}}{x_{2}-x_{1}})$$

given two data points ${(x}_{1},y_{1})$ and ${(x}_{2},y_{2})$ where $x_{1}$ and $x_{2}$ are the independent variable values, while $y_{1}$ and $y_{2}$ correspond to them. Equation (4) reflects the slope–intercept form of a line where $\left(\frac{y_{2}-y_{1}}{x_{2}-x_{1}}\right)$ represents the slope, and $\left(x-x_{1}\right)$ indicates the horizontal distance from $x_{1}$ to the new point x. By employing interpolation techniques, a complete time series dataset was obtained, enabling a more robust and comprehensive analysis.

### 3.2. Mutual Information Concept

The concept of mutual information (MI) was introduced by Shannon [33], although the term was first used by Fano [34]. MI is an element of probability theory and also information theory. On the ground of probability theory, MI is a measure of the interdependence of random variables. On the ground of information theory, MI is understood as shared information between two random variables. It can also be considered a measure of the stored information in one variable about another or the measure of the degree of predictability of the output variable knowing the input variabIe.

It is possible to determine MI for continuous variables as well as discrete variables. For two continuous variables X and Y, the mutual information $I\left(X;Y\right)$ is defined as [35]:

(5) $$I\left(X;Y\right)=\int_{y}^{}\int_{x}^{}P_{\left(X,Y\right)}\left(x,y\right)\log\left(\frac{P_{\left(X,Y\right)}\left(x,y\right)}{P_{X}\left(x\right)P_{Y}\left(y\right)}\right)dxdy$$

where $P_{\left(X,Y\right)}\left(x,y\right)$ is the joint probability density function of X and Y, $P_{X}\left(x\right)$ is the marginal probability density function of X, and $P_{Y}\left(y\right)$ is the marginal probability density function of Y.

Mutual information is a measure of the inherent dependence expressed in the joint distribution of X and Y relative to the marginal distribution of X and Y. It determines how different the joint probability distribution of the pair $\left(X,Y\right)$ is from the product of the marginal distributions of X and Y. On the ground of probability theory, if the variables X and Y are independent, their joint distribution is the product of their marginal distributions. In the opposite case, the variables are not independent. For independent variables, the mutual information is zero, $I\left(X;Y\right)=0$ (because $log\left(1\right)=0$). The MI = 0 indicates there is no information shared by X and Y. Zero is the minimum value of MI. For the dependent variables, MI is positive, and its value tells the amount of information shared. Most frequently, shannon is used as a unit of MI. In that case, the logarithm base in Equation (5) is 2. Mutual information is symmetric.

MI is a general metric. It is not limited to linear dependence like the correlation coefficient. MI analysis is capable of revealing both linear and non-linear associations in data. Generalizations of mutual information for more than two random variables have been developed.

### 3.3. Mutual Information Application

We proposed to apply mutual information analysis as a method to explore the co-dependence of indoor conditions in the parts of open spaces that are meant to act as functionally different zones. Open-kitchen restaurants with a kitchen and dining area were chosen as an example.

Indoor conditions could be described quantitatively with multiple measurable parameters. They included temperature, relative humidity, and various air compounds. In this study, we focused on the concentration of carbon dioxide and the total content of volatile organic compounds. They were selected as indicators that significantly contribute to explaining air quality in the kitchen and dining areas. The parameters can be easily and simply measured using the sensor technique.

To explore the co-dependence of indoor conditions between the kitchen zone and the dining zone, mutual information was calculated using measurement data on s T, RH, CO_2_, and TVOC collected in these two zones. We considered several kinds of mutual information.

- Mutual information for the parameter itself in one zone was determined using the general formula given by Equation (5), which was adapted as follows:

(6) $$MI\left(V_{i};V_{i}\right)=\int_{v_{i}}^{}\int_{v_{i}}^{}P_{\left(V_{i},V_{i}\right)}\left(v_{i},v_{i}\right)\log\left(\frac{P_{\left(V_{i},V_{i}\right)}\left(v_{i},v_{i}\right)}{P_{V_{i}}\left(v_{i}\right)P_{V_{i}}\left(v_{i}\right)}\right)dv_{i}dv_{i}$$

where V is the air parameter (T, RH, CO_2,_ or TVOC), and i is the indicator of the zone where the parameter was measured, either kitchen (i = 1) or dining zone (i = 2). For zone 1, we calculated $MI\left(T_{1};T_{1}\right)$, $MI\left({RH}_{1};{RH}_{1}\right),$ $MI\left({CO2}_{1};{CO2}_{1}\right),$ and $MI\left({TVOC}_{1};{TVOC}_{1}\right)$. Analogical calculations were performed for zone 2. $MI\left(V_{i};V_{i}\right)$ represents all information contained in the time series of parameter *V* in location *i*. Hence, it is the maximum information that the data on parameter V collected in location *i* can share.

- Mutual information between the parameter in one zone and the same parameter in another zone was determined using the general formula given by Equation (5), which was adapted as follows:

(7) $$MI\left(V_{1};V_{2}\right)=\int_{v_{2}}^{}\int_{v_{1}}^{}P_{\left(V_{1},V_{2}\right)}\left(v_{1},v_{2}\right)\log\left(\frac{P_{\left(V_{1},V_{2}\right)}\left(v_{1},v_{2}\right)}{P_{V_{1}}\left(v_{1}\right)P_{V_{2}}\left(v_{2}\right)}\right)dv_{1}dv_{2}$$

where $V_{i}$ is the air parameter measured in one zone (e.g., i = 1 kitchen), and $V_{j}$ is the air parameter measured in another zone (e.g., dining j = 2). We calculated $MI\left(T_{1};T_{2}\right),$ $MI\left({RH}_{1};{RH}_{2}\right),$ $MI\left({CO2}_{1};{CO2}_{2}\right),$ and $MI\left({TVOC}_{1};{TVOC}_{2}\right)$. $MI\left(V_{1};V_{2}\right)$ represents the information that is common for the time series of parameter V measured in location 1 and in location 2. Hence, $MI\left(V_{1};V_{2}\right)\leq MI\left(V_{1};V_{1}\right)$, and also $MI\left(V_{1};V_{2}\right)\leq MI\left(V_{2};V_{2}\right)$.

- Scaled mutual information was determined using the following formula:

(8) $${MI}_{V}=\frac{MI\left(V_{1};V_{2}\right)}{max\left(MI\left(V_{1};V_{1}\right),MI\left(V_{2};V_{2}\right)\right)}$$

The scaled mutual information tells, in terms of fraction, the part of the overall information contained in the time series of air parameters measured in one zone that is shared with the same parameters in another zone. We calculated MI_T_, MI_RH_, MI_CO2_, and MI_TVOC._

While $MI\left(V_{1};V_{2}\right)$, $MI\left(V_{1};V_{1}\right),$ and $MI\left(V_{2};V_{2}\right)$ are simply nonnegative, ${MI}_{V}$ belongs to the range between zero and one. ${MI}_{V}\in \langle 0,1\rangle$ allows for convenient comparison of the degree of co-dependence between indoor conditions in the kitchen and the dining zones regarding various air parameters and in various objects. Further in this work, when using the abbreviation MI, we are referring to scaled mutual information MI_v_ as defined by Equation (8) without pointing at the particular air parameter.

### 3.4. Mutual Information Computational Algorithm

We calculated the mutual information score using the k-Nearest Neighbor (k-NN) method, applying a sliding window approach to a dataset comprising 300 windows (N = 300). The computation process was conducted using Python language. The procedure involved several steps. First, we defined a distance metric using the Euclidean distance formula to measure the distances between data points. Then, for each data point ($x_{i},\;y_{i}$), we selected the three nearest neighbors (k = 3) based on the distance metric, resulting in a set of k-NN $N_{k}(x_{i},y_{i})$ for each data point. We calculated the volume of the 3-dimensional hypersphere ${(V}_{3(r_{i})})$ using the appropriate formula. Next, we counted a normalization constant C to ensure that the estimated joint probability density function (PDF) integrated to one over the entire domain of the variables. This normalization step is crucial for maintaining the consistency and interpretability of the mutual information as a measure of shared information between the variables [31].

(9) $$V_{3\left(r_{i}\right)}=\left(\frac{4}{3}\right)pi{\left(r_{i}\right)}^{3}$$

(10) $$C=N/(\sum_{\left\{i=1\right\}}^{N}\left(kV_{3\left(r_{i}\right)}\right))$$

The k-NN method leverages the local structure of the data by estimating the joint probability function considering neighborhood information, hypersphere volume, and normalization for each individual data point ($x_{i},\;y_{i}$). This estimation enables us to effectively capture the local structure and density surrounding each data point. We estimated the joint probability density function ${f(x}_{i},\;y_{i})$ using the provided equation [36].

(11) $$f\left(x_{i},y_{i}\right)=C/(kV_{3\left(r_{i}\right)})$$

The k-NN method relies on the concepts of entropy and joint entropy. Hence, we computed the joint entropy $\left(H\left(X,Y\right)\right)$ and the marginal entropies $H\left(X\right)$ and $H\left(Y\right)$ using the respective Equations (12)–(15). The joint entropy quantifies the level of uncertainty or randomness present in the simultaneous values of the two variables [36,37].

(12) $$H\left(X,Y\right)=-\int \int f\left(x,y\right)\log\left[f\left(x,y\right)\right]dx\;dy$$

(13) $$H\left(X\right)=-\int f\left(x\right)\log\left[f\left(x\right)\right]dx$$

(14) $$H\left(Y\right)=-\int f\left(y\right)\log\left[f\left(y\right)\right]dy$$

(15) I(X;Y) = H(X)+H(Y)−H(X,Y)

Finally, we obtained the mutual information score I(X; Y) using Equation (15) by considering the entropies of X (H(X)) and Y (H(Y)), as well as the joint entropy H(X, Y). The mutual information score provides a measure of the information shared between the variables considering both individual and combined uncertainties or information content [36,37,38].

### 3.5. Mutual Information Analysis

In the zones of open space, indoor conditions and their co-dependence are mainly influenced by the external sources of mass and heat, the internal sources of mass and heat, as well as the mass and heat exchange between zones. The processes of heat and mass exchange between zones are intensified by air mixing. Due to the lack of physical barriers between zones, the air airflow is not restricted.

It was assumed that the interdependence between IAQ in zones could change during the day, in particular, when comparing the hours of restaurants’ operation with the time when they were closed. The characteristic functions of the kitchen and restaurant zones are revealed during the operation of the facility and cease when it is closed. Thus, over a typical day, at least these two periods could differentiate the zones’ impact on the indoor environment, both their own and the other zones. During working hours, sources of CO_2_, such as people and combustion processes (for cooking and frying), appear in the restaurant. At the same time sources of TVOC become active. They come from the processes of food preparation, the exposure of food during consumption, and cleaning activities. Heat sources get triggered during business hours. They are needed for cooking, frying, grilling, and baking processes. People and solar radiation are heat sources as well. Water vapor comes from the processes of washing, cooking, wiping tables and floors, as well as the metabolism of people. Also, the properties of the ventilating air affect the studied air parameters. Sources of CO_2_, TVOC, water vapor, and heat are present in both zones but do not act with equal intensity and in the same way. At night, sources of mass and heat are inactive indoors. The entire space is influenced by outdoor conditions and TVOC desorption from furnishing. During business hours, it is desirable to ensure appropriate indoor conditions in each zone. At nighttime, such activities can be significantly reduced or stopped.

It should be noted that the co-dependence of conditions in two zones is not the same as the similarity of conditions. In the general, there may be different conditions in co-dependent zones. Significant interdependence means so much that, knowing the conditions in one zone, it is possible to determine with high probability the conditions in another zone on this basis using the appropriate mathematical model of interdependence. In such cases, the monitoring of air parameters can be limited to one zone and supplemented with models of air parameter interdependence between zones. The validity of this approach as an alternative to multi-zone monitoring depends on the strength of the interdependencies and their stability over time.

For this reason, this paper presents an analysis of MI from a long-term and short-term perspective. Long-term MI was calculated for two datasets. One was associated with restaurant operating hours, and the other was linked to the time when the restaurant was closed. Short-term MI was calculated for data in the time window of 10 min. The order processing duration in the restaurant is approximately 10 min. Potentially, this period is sufficient to cover the entire spectrum of conditions related to the realization of this basic unit process in the kitchen area. Order processing influences the conditions in the facility in the most varied yet intense way. The MI analysis performed with this time resolution covers these mutual interactions for which the time lag between the occurrence of the stimulus in one zone and the effect in the other does not exceed 10 min. The sliding time window technique was employed to determine MI in the entire measurement period.

## 4. Results

### 4.1. Two-Dimensional Frequency of Occurrence Histograms for Air Parameters in Two Zones of Open Kitchens

**Figure 3:**
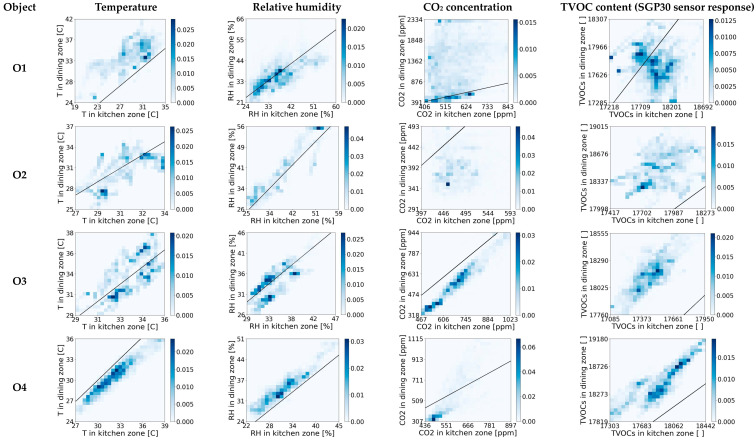
Two-dimensional histograms of frequency of occurrence for air parameters (T, RH, CO_2_, and TVOC) in two zones of open-kitchen restaurants (O1, O2, O3, O4) during business hours.

**Figure 4:**
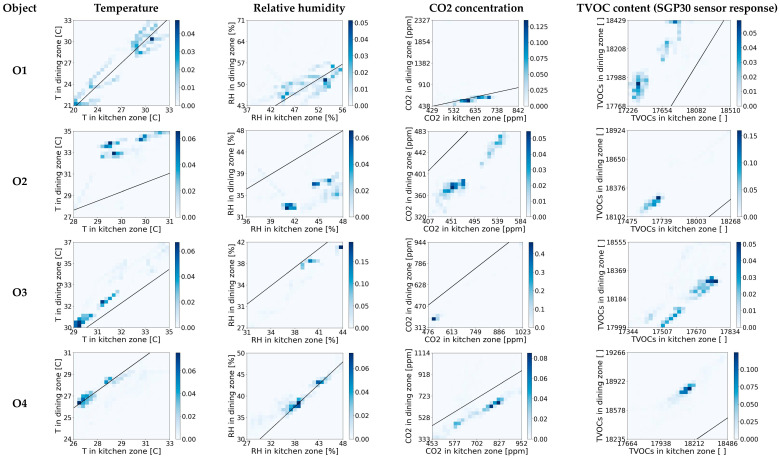
Two-dimensional histograms of frequency of occurrence for air parameters (T, RH, CO_2_, and TVOC) in two zones of open-kitchen restaurants (O1, O2, O3, O4) at the time when the restaurants were closed.

A qualitative analysis of the interdependence of the indoor air parameters between the kitchen and the dining zones in open-kitchen restaurants was carried out first. It was based on two-dimensional frequency histograms. An individual histogram presents the frequency of occurrence of one air parameter in the two zones. Histograms for the business hours are shown in Figure 3. Analogous histograms for the time when the restaurants were closed are shown in Figure 4. Based on Figure 3 and Figure 4, the indoor conditions interdependence between zones was different depending on the air parameter and the facility under consideration. An example of a histogram representing the lack of interdependence was the one for the TVOC in O1 during working hours (Figure 3). For the predefined level of TVOC in the kitchen zone, the associated TVOC content in the restaurant zone was poorly defined, i.e., a wide range of values was possible. examples of histograms representing clear interdependencies, refer to the T and RH in O6 at the time when the restaurant was closed (Figure 4). In this case, it is possible to indicate the narrow range of the most likely occurring T and RH values in the restaurant zone when the level of these parameters in the kitchen zone is defined. A comparison of Figure 3 and Figure 4 shows that all air parameters in the zones were more interdependent at nighttime (Figure 4) compared to working hours (Figure 3). The observed correlations were positive; i.e., higher values of air parameters in one zone were associated with higher values in another zone. In general, the CO_2_ concentration and TVOC content were higher in the kitchen area than in the dining zone. This was the case in all facilities both when the restaurants were in operation (Figure 3) and when they were closed (Figure 4). In contrast, the behavior of the temperature and humidity was inconsistent. For example, in the O2 and O3 facilities, during the restaurant’s operating time, the temperature was similar in both zones and so was the RH. After closing, the conditions in the zones became increasingly different. The dining zone became warmer and drier than the kitchen zone. The opposite situation occurred in facilities O1 and O4. During business hours, the T and RH in each zone were different, and after closing, their levels equalized. The analysis of the histograms in Figure 3 and Figure 4 shows that the long-term interdependencies of the chemical air parameters, such as the CO_2_ and TVOC concentrations, developed differently in the open-kitchen restaurants than the co-dependence of the thermodynamic parameters, like the T and RH.

### 4.2. IAQ Interdependence between Functionally Different Zones of Open-Kitchen Restaurants

For the quantitative analysis of the IAQ interdependence between the kitchen and the dining zones in the open-kitchen restaurants, mutual information was used. Based on the properties of MI and utilizing its synthetic, numerical form, the interdependencies were compared for several air parameters of the four facilities. The temporal variation of co-dependencies was examined. Long-term and short-term interdependencies were explored.

#### 4.2.1. Long-Term MI Associated with Business Hours and the Time When the Restaurants Were Closed

**Figure 5:**
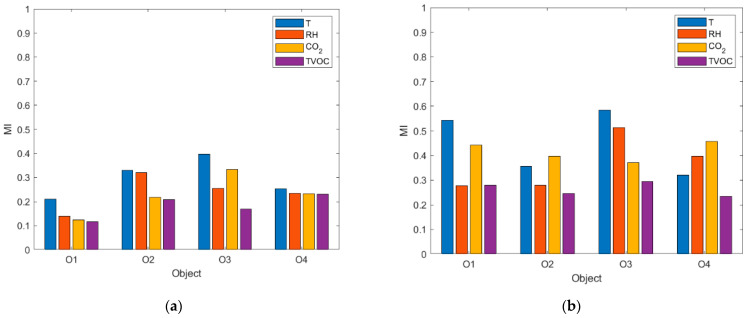
Mutual information (MI) between air parameters (T, RH, CO_2,_ and TVOC) in the kitchen and dining zones in four open-kitchen restaurants (O1, O2, O3, and O4) based on a set of measurement data relating to (**a**) the time when the restaurant was in operation, (**b**) the time when the restaurant was closed. Long-term approach.

Figure 5 shows the MI between the IAQ in the kitchen and restaurant zones when the restaurants were in operation (Figure 5a) and when the restaurants were closed (Figure 5b) using a long-term perspective. During operating hours, the MI ranged from 0.1172 to 0.3979 (Figure 4a). Thus, between 12% and 40% of the information about one zone and contained in the entire time series of one of the analyzed air parameters referred to the other zone at the same time. This percentage was relatively small, indicating that conditions in the zones were weakly interdependent during business hours. When the restaurants remained closed, the MI was higher, ranging from 0.2351 to 0.5842 (Figure 5b). The time series of air parameters in the zones shared between 24 and 58%, indicating that outside business hours, the interdependence of the conditions in the zones was slightly higher than during operating hours.

As Figure 5 shows, the MI took on different values depending on the air parameter. During restaurant operation hours, MI_T_ was highest (Figure 5a). MI_RH_ was comparable to MI_CO2_ and slightly smaller than MI_T_ (except at O3). The lowest values were found for MI_TVOC_. The following ranking: MI_T_ > MI_RH_ > MI_CO2_ > MI_TVOC_ indicates that during the restaurant’s operation, the co-dependence of temperature was the greatest, CO_2_ and RH were slightly less co-dependent, and the co-dependence of TVOC was the smallest. Slightly different relationships occurred outside working hours (Figure 5b). At this time, MI_T_ and MI_CO2_ were the highest. MI_RH_ took relatively smaller values. MI_TVOC_, on the other hand, remained the smallest, as it did during business hours. Thus, when the restaurant was not operating, the interdependence of temperature in the zones was still high but comparable to the CO_2_ concentration. The interdependence of relative humidity between zones was noticeably lower and similar to the TVOC. This one was the smallest. Despite the identified general regularities, the proportions of MI_T_, MI_RH_, MI_CO2,_ and MI_TVOC_ varied slightly across facilities. An element of facility specificity is evident from the results of the analysis.

The results of the quantitative analysis of the air parameter interdependence using MI correspond to the results of the qualitative analysis based on the 2D histograms (compare Figure 5a with Figure 3 and Figure 5b with Figure 4). In both cases, long-term MI was examined.

#### 4.2.2. Short-Term MI Associated with Business Hours and the Time When the Restaurants Were Closed

**Figure 6:**
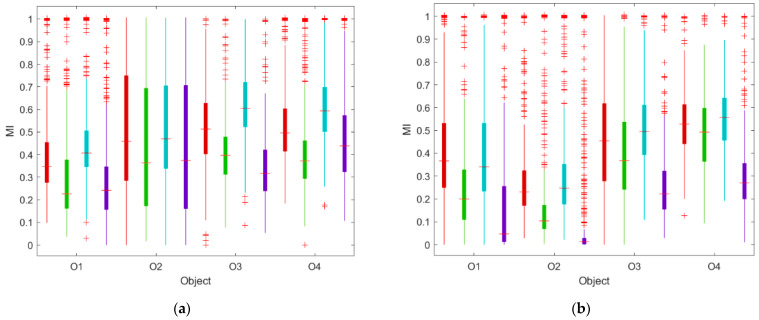
Mutual information between air parameters (T, RH, CO_2_, and TVOC) in the kitchen and restaurant zones in four open-kitchen restaurants (O1, O2, O3, and O4) based on a set of measurement data relating to (**a**) the time when the restaurant was in operation, (**b**) the time when the restaurant was closed. Short-term approach. Color-coded are the results related to individual air parameters: red—T, green—RH, marine—CO_2_, purple—TVOC.

Figure 6 shows the statistics of the short-term MI between the kitchen and restaurant zones when the restaurants were in operation (Figure 6a) and when the restaurants were closed (Figure 6b). The statistics are displayed using boxplots. The medians of MI_T_, MI_RH_, MI_CO2,_ and MI_TVOC_ are shown additionally in Table 3.

The short-term approach revealed a consistent ranking of MIs, which was valid in all facilities during both operating and closing hours. The highest was MI_CO2_ (Figure 6, marine boxplot; median: 0.247–0.556). Slightly lower values occurred for MI_T_ (Figure 6, red boxplot; median: 0.230–0.529). MI_RH_ was smaller (Figure 6, green boxplot; median 0.104–0.492). MI_TVOC_ was the smallest (Figure 6, violet boxplot; median 0.013–0.270). As shown in Figure 6 and Table 3, the operation of the facilities caused a change in MIT_VOC_ and MI_CO2_, but it affected MI_T_ and MI_RH_ to a lesser extent. For MI_TVOC_, significantly higher values were found when the facility was in operation (median: 0.243–0.439) than when it was closed (median: 0.047–0.270). This occurred in all restaurants. Also, MI_CO2_, in all restaurants, was higher during operating hours (median: 0.407–0.593) than when the restaurants were closed (median: 0.247–0.556). The MI_T_ change between restaurant operating hours (0.347–0.513) and outside them (0.230–0.529) was relatively smaller and inconclusive. In two facilities (O2 and O3), the temperature co-dependence was higher during the operating time, and in two others (O1 and O4), the opposite held true. Similarly, MI_RH_ was not significantly different during restaurant operating hours (median 0.227–0.397) or outside them (median 0.104–0.492). In three of the four restaurants (O1, O2, and O3), the MI was higher during operating hours.

The results of the MI analysis in the short-term (Figure 5) and long-term (Figure 4) perspectives are different. On average, long-term MI_T_, MI_RH_, MI_CO2,_ and MI_TVOC_ were smaller than their short-term counterparts. The analysis of the MI on a long-term basis indicated that all air parameters were more co-dependent when the restaurant was closed compared to operating hours. In the short-term, the restaurant functioning had an impact on the interdependence of the chemical air parameters, i.e., the TVOC and CO_2_ concentrations, while the interdependence of the thermodynamic air parameters, the T and RH in the zones, did not differ significantly during restaurant operation and closure. In both periods, the short-term MI_T_, MI_RH_, MI_CO2,_ and MI_TVOC_ were characterized by a very large spread of values.

#### 4.2.3. Diurnal Variation of MI_T_, MI_RH_, MI_CO2_, and MI_TVOC_

**Figure 7:**
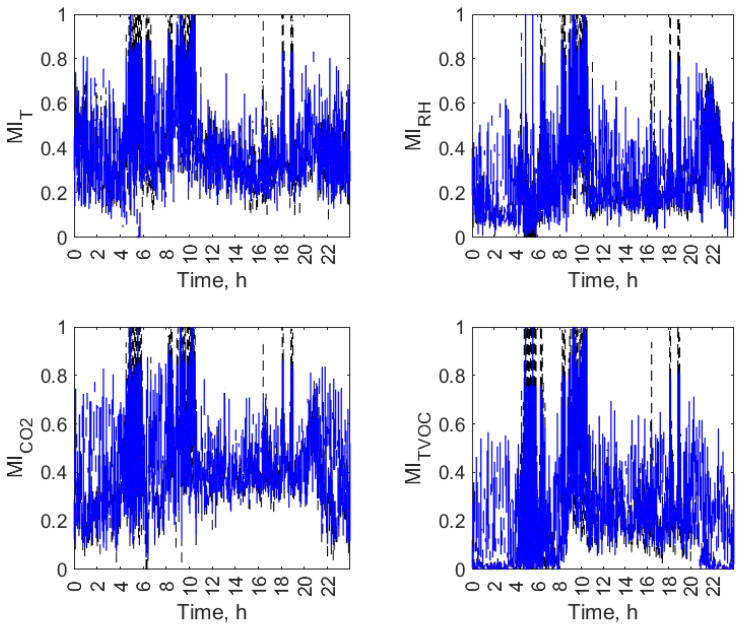
Daily variation of mutual information (MI) between air parameters (T, RH, CO_2_, and TVOC) in the kitchen and restaurant zones of open-kitchen restaurant, O1.

**Figure 8:**
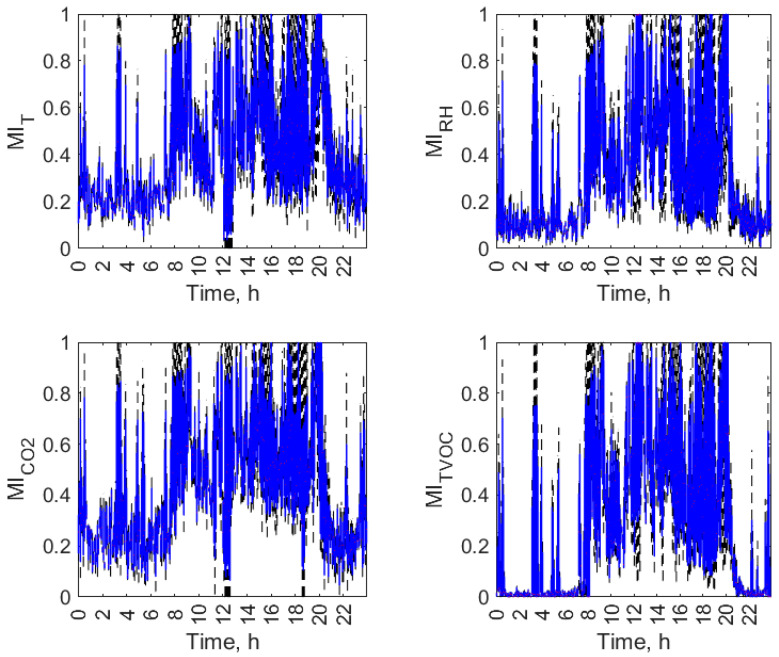
Daily variation of mutual information (MI) between air parameters (T, RH, CO_2_, and TVOC) in the kitchen and restaurant zones of open-kitchen restaurant, O2.

**Figure 9:**
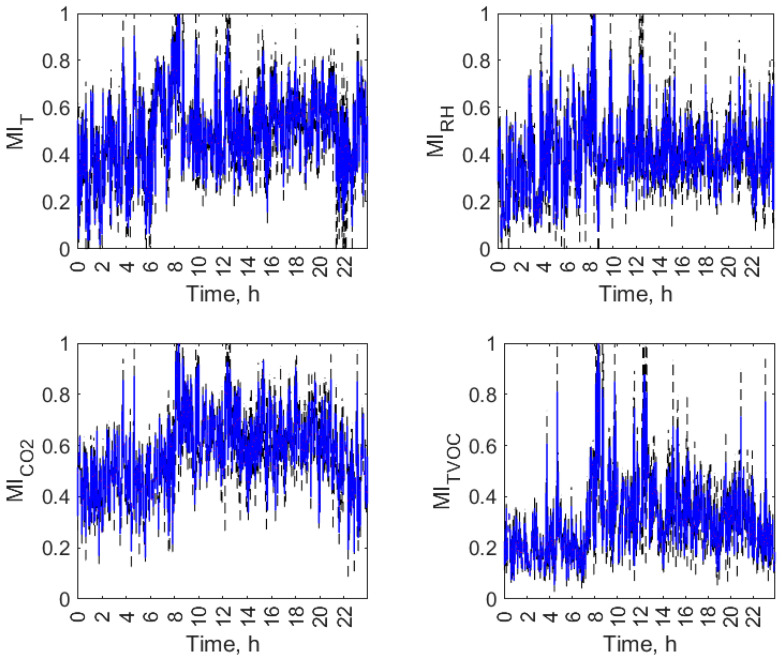
Daily variation of mutual information (MI) between air parameters (T, RH, CO_2_, and TVOC) in the kitchen and restaurant zones of open-kitchen restaurant, O3.

**Figure 10:**
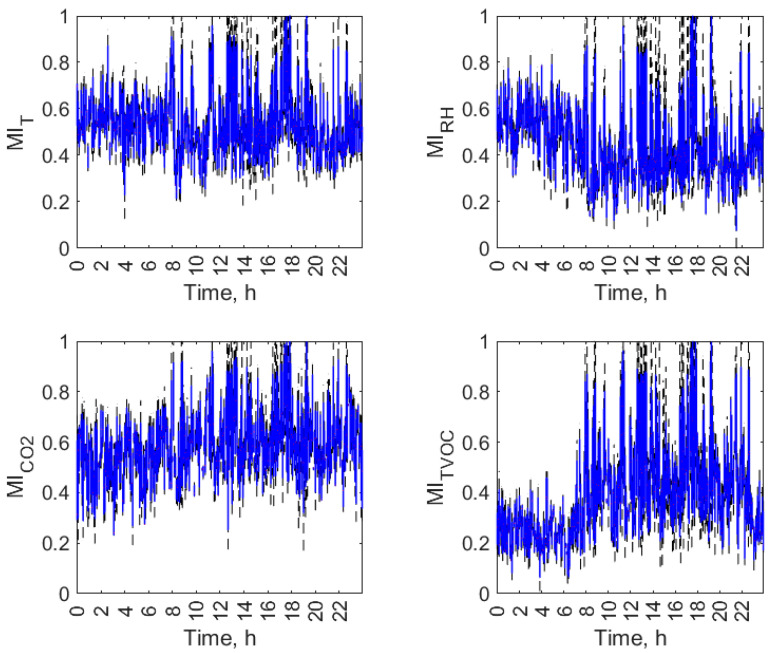
Daily variation of mutual information (MI) between air parameters (T, RH, CO_2_, and TVOC) in the kitchen and restaurant zones of open-kitchen restaurant, O4.

Figures from Figure 7, Figure 8, Figure 9 and Figure 10 display the daily variation of short-term MI for all air parameters in the individual open-kitchen restaurants: O1 (Figure 7), O2 (Figure 8), O3 (Figure 9), and O4 (Figure 10). As shown, the variation in the MI and therefore the changes in the interdependence of the conditions in the kitchen and dining zones over a day were not exclusively related to the status of the facility (operation or non-functioning). The dynamics of temporal changes were greater. There was a morning increase in MI values at the start of the restaurant’s operation. Depending on the facility, it was observed between 5 a.m. (O1) and 8 a.m. (O2, O3 and O4). In all objects, this effect was evident for the TVOC, and in three facilities (O1, O2, and O3), it was observed for all air parameters. There was a decrease in MI values during restaurant closing time. It was observed between 8 p.m. (O2) and 10 p.m. (O1, O3, and O4) depending on the facility. In all facilities, an evening decrease in MI was found for the TVOC and CO_2_. For the other parameters, it was observed in O1 (Figure 7) and O2 (Figure 8). The additional increase in MI was noticed in the afternoon and evening hours in O1 (from 8:00 p.m. to 9:00 p.m.) and O2 (from 4:00 p.m. to 8:00 p.m.). The morning and afternoon MI increase as well as the evening MI decrease can be linked to changes in restaurant operations. The start of work and peak hours are associated with an increased activity of emission sources in both zones (equipment and people) and improved air mixing conditions between zones (customer movement, service movement), which can account for the increase in the interdependence of conditions in the zones. The opposite effect occurs when the restaurants are closed. In addition to the three aforementioned effects seen in multiple restaurants, the MI had its dynamics of change in each facility.

#### 4.2.4. Correlation of Short-Term MIs for Different Air Parameters

**Figure 11:**
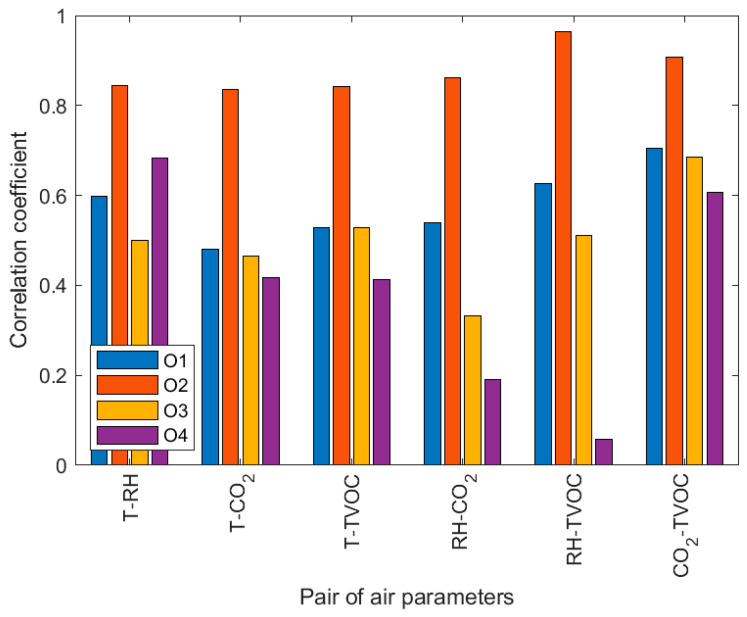
The correlation coefficient between short-term MIs for different air parameters in the same facility.

Figure 11 shows the correlation coefficient between the short-term values of MI_T_, MI_RH_, MI_CO2,_ and MI_TVOC_ in the four open-kitchen restaurants.

High correlation coefficients were found between MI_CO2_ and MI_TVOC_ (R = 0.6058 – 0.9052). Slightly smaller but also high were the correlation coefficients in the case of MI_T_ and MI_RH_ (R = 0.5007 – 0.8441). Weaker correlations were between MI_T_ and MI_CO2_ (R = 0.4175 – 0.8357) as well as between MI_T_ and MI_TVOC_ (R = 0.4122 − 0.8419). The correlations between MI_RH_ and MI_CO2_ (R = 0.1911 − 0.8614) as well as between MI_RH_ and MI_TVOC_ (R = 0.0571 − 0.9642) were still lower, but highly diverse in different restaurants. The analysis revealed several regularities regarding the correlation of the temporal changes in the indoor condition interdependence between zones. The MI correlations were high for the parameters indicating the chemical quality of the indoor air (MI_CO2_ vs. MI_TVOC_). In other words, the increasing co-dependence between zones regarding the CO_2_ concertation was associated with the increasing co-dependence regarding the content of volatile organic compounds in the indoor air. The MI correlations were also high for the parameters indicating thermal conditions (MI_T_ vs. MI_RH_). That is, when the co-dependence of the zones’ temperatures increased, there also increased the co-dependence of the zones’ humidity. However, when looking at the air parameters representing the chemical air quality vs. the parameters representing the thermal conditions, the MI correlations were smaller. The correlations of MI_T_ vs. MI_CO2_ and MI_T_ vs. MI_TVOC_ were weak and the correlations of MI_RH_ vs. MI_CO2_ and MI_RH_ vs. MI_TVOC_ were additionally highly diverse among facilities.

## 5. Discussion

This paper presents an analysis of the interdependence of indoor conditions in zones of different functionalities in open spaces using the example of an open-kitchen restaurant. The practical dimension of this work is proposing an approach for assessing the validity and scope of multi-zone monitoring of indoor air parameters as a source of information for ventilation systems for maintaining appropriate conditions in these zones.

MI analysis was applied to quantitatively examine the interdependence between the air parameters in the kitchen and dining zones. Measurements of the T, RH, CO_2_, and TVOC were conducted using sensor technology. MI calculations were made from a long-term and short-term perspective. The IAQ interdependence was explored regarding (1) the time perspective, (2) individual air parameters, (3) the open-kitchen restaurants included in this study, and (4) time as a proxy for the diurnal rhythm of restaurant activity.

The results of this analysis depended on the length of the data time series taken as the basis for the MI calculations. Long-term MI was generally small and smaller than the average short-term MI in the same period (business hours or the time when the restaurant was closed). This result calls into question building statistical models based on long-term intercorrelations to be able to apply single-zone monitoring for multi-zone ventilation control. Such models would exhibit low performance and unsuitability for providing comprehensive information for indoor ventilation control at a high temporal resolution. This particularly holds for working hours when control of the indoor conditions is needed. Short-term models could be more effective, especially since short-term MI tended to be higher during restaurant operating hours than outside them. However, short-term MI was characterized by high temporal variability and a large spread of values. These facts put into question the building of statistical models based on short-term intercorrelation to be able to apply single-zone indoor air monitoring for multi-zone ventilation control. Overall, the obtained results encourage the multi-zone monitoring of air parameters in open-kitchen restaurants.

This study shows that the interdependence of IAQ in the zones depended on which air parameter was considered. Thus, a comprehensive assessment of the interdependence of indoor conditions requires performing an analysis for each air parameter separately. Based on our study, the lowest and objectively low MI values occurred for the TVOC (median MI during restaurant hours, 0.243–0.439). Controlling indoor conditions regarding this parameter in open-kitchen restaurants shall be based on the continuous measurement in each zone. The matter is really important because TVOCs are related to the odorous aspect of indoor air quality perception. In particular, in facilities of the type under consideration, this issue is crucial. The highest short-term MI during business hours was found for the CO_2_ concentration (median MI, 0.407–0.593). This is the only air parameter among those studied that could be possibly measured in one zone and determined in the other zone using an appropriate mathematical model.

Based on the analysis of the short-term MI, the interdependence of the air parameters in the zones was subject to temporal variability. This variability can be linked to the rhythm of restaurant activity, more precisely, to the varying conditions of air exchange between zones as well as the presence and functioning of mass or heat sources. In particular, elevated MI values were found at the times of starting work, ending work, and during periods of increased customer traffic. The instantaneous increase in MI appeared related with busy hours and associated with the enhanced air mixing. The most pronounced changes in the MI related to restaurant operations were observed for the TVOC. The interdependence of the IAQ in zones with respect to this parameter was subject to the largest and most dynamic changes.

This study showed that the interdependence of individual air parameters in the zones, while subject to general regularities common to all open-kitchen restaurants, also showed variation across sites. The level of MI was not a function of the distance between the measurement points. Small MI values were not associated with large distances between sensing devices, as in sites O2 (8.4 m) and O4 (6 m). Large MI values occurred when the multi-sensor devices were located quite close to each other, as in sites O3 (2 m) and O1 (3.6 m). The specificity of the IAQ interdependencies was related to the arrangement of the facility and the way it was functioning, in a broad sense. The general features of the IAQ interdependence detected in this work could be expected in any open-kitchen restaurant. However, the actual strength of the interdependencies as well as their variability over time can be considerably different.

The CO_2_ concentration and TVOC content are considered representative of chemical air quality, while the temperature and relative humidity are considered descriptors of the thermal conditions indoors. The correlation analysis of the MI values for these air parameters within a single facility showed that the correlations were highest between MI_CO2_ and MI_TVOC_ as well as between MI_T_ and MI_RH._ On the other hand, weak and highly object-specific correlations between MI_T_ and MI_CO2_, MI_T_ and MI_TVOC,_ as well as between MI_RH_ and MI_CO2_, MI_RH_ and MI_TVOC_ were revealed. The analysis of the correlation between short-term MI_T_, MI_RH_, MI_CO2,_ and MI_TVOC_ draws attention to the discrepancy between the temporal evolution of the co-dependence of the thermal conditions and chemical air quality in the kitchen zone and the dining zone of an open-kitchen restaurant. It shows that these two groups of parameters convey different information about indoor conditions’ co-dependence in multi-zone objects. Therefore, at least one parameter from each group shall be monitored to attain a comprehensive picture of the indoor condition co-dependence in a multi-zone object.

Our approach can be used to comprehensively analyze the co-dependence of IAQ in other open-space facilities with functionally different zones. In general, they may develop differently than in open-kitchen restaurants.

## 6. Conclusions

This paper presents an analysis of indoor condition interdependence between the kitchen and dining zones in open-kitchen restaurants. The approach was based on the mutual information analysis of indoor air measurement data on the T, RH, CO_2,_ and TVOC collected using sensor technology.

Based on this study, some general features of the IAQ interdependence in the kitchen and dining zones in open-kitchen restaurants can be identified.

The degree of interdependence was parameter-dependent. The weakest mutual dependence between zones was found for the TVOC (short-term and long-term). The strongest co-dependence existed for the CO_2_ (short-term) and T (long-term).

The interdependence of indoor air parameters was generally low (MI less than 0.6). The long-term interdependencies were weaker than the short-term ones. The ranking of long-term MI values for the individual air parameters was MI_CO2_ (0.34) ≅ MI_T_ (0.34) > MI_RH_ (0.28) > MI_TVOC_ (0.23), and the ranking of short-term MI values was MI_CO2_ (0.48) > MI_T_ (0.46) > MI_RH_ (0.37) > MI_TVOC_ (0.26).

The short-term interdependencies were subject to temporal variability, which could be linked to the daily rhythm of restaurant activity. Stronger short-term interdependencies between zones occurred during business hours, in particular, at the times of starting work, ending work, and during periods of increased customer traffic. The increased co-dependence could be associated with increased air mixing between zones.

It was found that the interdependencies of various indoor air parameters in the same object behaved differently. In particular, the analysis showed a distinct behavior of co-dependence between the zones regarding the thermal conditions (indicated with MI_T_ and MI_RH_) and chemical air quality (indicated with MI_CO2_ concentration and MI_TVOC_ content). Therefore, the notion of the co-dependence of IAQ between zones is not internally coherent. It is more appropriate to address the interdependence of individual air parameters.

Despite the detected general regularities, the strength of IAQ interdependencies as well as their temporal variability can be considerably different for individual open-kitchen restaurants.

The small level, high temporal variation, and object specificity of the co-dependencies of the air parameters between the kitchen and dining zones provide arguments for the multiparametric, multi-zone monitoring of air parameters as the most reliable source of information on air quality control in open kitchens.

The approach presented in this work may be applied to examine the co-dependence between functionally different zones in any interiors given the design of indoor air quality monitoring and ventilation systems, assuring a high quality of indoor air.

## Figures and Tables

**Table 1:** Basic information concerning open-kitchen restaurants involved in this study.

Object	Type of Restaurant	Room Size	Business Hours	Monitoring Period	Distance between Measurement Points [m]	Location and Surrounding	Meat Preparation Method	Plate Used	Estimated Number of Customers per Day
**O1**	Take away	2 m × 3 m × 2 m	9:00–22:00	10–16 June 2021	3.6	City center; kitchen zone facing the pedestrian area; dining zone facing tram and bus stop	The employee served the heated meats. He filled the meat heater with the grilled meats. The grilling process took approximately 15–20 min depending on the day.	Disposable plates	60–70
**O2**	Quick service	7 m × 6 m × 2.5 m	9:00–22:00	18–22 June 2021	8.4	Suburb; kitchen zone facing backyard; dining zone facing the street	The meats were grilled after the customer made orders.	Ceramic plates and sauce containers.	100–140
**O3**	Quick service	6 m × 6 m × 2.5 m	10:00–22:00 (weekday) 10:00–23:00 (weekend)	11–15 August 2021	2.2	Near train station; kitchen zone facing residential area; dining zone facing the street	Same as Object 1	Disposable plates	60–70
**O4**	Quick service	7 m × 5 m × 2.3 m	10:00–22:00 (weekday) 10:00–00:00 (weekend)	16–21 August 2021	6	Residential area; kitchen zone and dining zone facing the residential area	Same as Object 1	Disposable plate	40–50

**Table 2:** The detailed characteristics of sensors mounted in a multi-sensor device. The sensors measured air parameters analyzed in this work [30].

Sensor	Measured Parameter	Detection Principle	Measurement Range	Accuracy	Resolution	Repeatability	Long Term Drift
**SHT25**	Temperature	Bandgap temperature sensor	−40 to 125 °C	Typ. ±0.2 °C	0.04 °C	±0.1 °C	<0.02 °C/yr
	Relative humidity	Capacitive type humidity sensor	0 to 95%RH	±1.8%RH	0.04%RH	±0.1%RH	<0.25 %RH/yr
**SCD30**	CO_2_	Non-Dispersive Infrared (NDIR) measurement technology	0–5′000 ppm (PWM)	±(30 ppm + 3% meas. value)	−	±10 ppm	±50 ppm
**SGP30**	Total volatile organic compounds	Metal-oxide gas sensor (chemical resistors)	0.3 ppm to 30 ppm ethanol 0 ppm to 1000 ppm ethanol	Typ. 15% of meas. value	Typ. 0.2% of meas. value	−	Typ. 1.3% of meas. Value

**Table 3:** Median of MI for business hours (BH) and when the restaurants were closed (RC). Short-term approach.

Object	MI_T_		MI_RH_		MI_CO2_		MI_TVOC_	
	BH	RC	BH	RC	BH	RC	BH	RC
**O1**	0.347	0.367	0.227	0.200	0.407	0.341	0.243	0.047
**O2**	0.459	0.230	0.363	0.104	0.470	0.247	0.374	0.013
**O3**	0.513	0.454	0.397	0.368	0.605	0.495	0.317	0.222
**O4**	0.496	0.529	0.372	0.492	0.593	0.556	0.439	0.270

## Data Availability

The data are not publicly available and are available upon request from the owners of the premises where the measurements were performed.

## References

[1] Barber L.B., Münster M.B. (2022). Aspects of openness in Hong Kong coffee shops. Interiors.

[2] Byun J., Jang S. (2019). Can signaling impact customer satisfaction and behavioral intentions in times of service failure? Evidence from open versus closed kitchen restaurants. J. Hosp. Mark. Manag..

[3] Basu A. (2022). Pros and Cons of Open Kitchens in the Restaurant Industry. https://modernrestaurantmanagement.com/pros-and-cons-of-open-kitchens-in-restaurant-industry/.

[4] Nisbets Australia (2023). Why do Restaurants Have Open Kitchens?. https://www.nisbets.com.au/why-do-restaurants-have-open-kitchens.

[5] Byun J., Jang S. (2018). Open kitchen vs. closed kitchen: Does kitchen design affect customers causal attributions of the blame for service failures?. Int. J. Contemp. Hosp. Manag..

[6] Sohn E.-M., Lee K.-W. (2018). The effect of chefs’ nonverbal communication in open kitchens on service quality. J. Foodserv. Bus. Res..

[7] Graham D., Ali A., Tajeddini K. (2020). Open kitchens: Customers’ influence on chefs’ working practices. J. Hosp. Tour. Manag..

[8] Szczurek A., Azizah A., Maciejewska M. (2022). The Detection of Activities Occurring Inside Quick Service Restaurants That Influence Air Quality. Sensors.

[9] Chang H., Capuozzo B., Okumus B., Cho M. (2021). Why cleaning the invisible in restaurants is important during COVID-19: A case study of indoor air quality of an open-kitchen restaurant. Int. J. Hosp. Manag..

[10] Abdullahi K.L., Delgado-Saborit J.M., Harrison R.M. (2013). Emissions and indoor concentrations of particulate matter and its specific chemical components from cooking: A review. Atmos. Environ..

[11] Lee S.C., Li W.-M., Chan L.Y. (2001). Indoor air quality at restaurants with different styles of cooking in metropolitan Hong Kong. Sci. Total Environ..

[12] Taner S., Pekey B., Pekey H. (2013). Fine particulate matter in the indoor air of barbeque restaurants: Elemental compositions, sources and health risks. Sci. Total Environ..

[13] Cheng J.-H., Lee Y.-S., Chen K.-S. (2016). Carbonyl compounds in dining areas, kitchens and exhaust streams in restaurants with varying cooking methods in Kaohsiung, Taiwan. J. Environ. Sci..

[14] Arı A., Arı P.E., Yenïsoy-Karakaş S., Gaga E.O. (2020). Source characterization and risk assessment of occupational exposure to volatile organic compounds (VOCs) in a barbecue restaurant. Build. Environ..

[15] Lee H., Shuai J., Woo B., Hwang M.-Y., Park C.-H., Yu S.-D., Yang W. (2011). Indoor Air Quality at Restaurants and Bars in Evening Hours in Korea. Epidemiology.

[16] Poon C., Wallace L., Lai A.C. (2016). Experimental study of exposure to cooking emitted particles under single zone and two-zone environments. Build. Environ..

[17] Laarne P., Amnell E., Zaidan M.A., Mikkonen S., Nieminen T. (2022). Exploring Non-Linear Dependencies in Atmospheric Data with Mutual Information. Atmosphere.

[18] Ulpiani G., Hart M.A., Di Virgilio G., Maharaj A.M. (2021). Urban meteorology and air quality in a rapidly growing city: Inter-parameter associations and intra-urban heterogeneity. Sustain. Cities Soc..

[19] Hu D., Wu J., Tian K., Liao L., Xu M., Du Y. (2017). Urban air quality, meteorology and traffic linkages: Evidence from a sixteen-day particulate matter pollution event in December 2015, Beijing. J. Environ. Sci..

[20] Zaidan M.A., Haapasilta V., Relan R., Paasonen P., Kerminen V.-M., Junninen H., Kulmala M., Foster A.S. (2018). Exploring non-linear associations between atmospheric new-particle formation and ambient variables: A mutual information approach. Atmos. Chem. Phys..

[21] Chen S., Kan G., Li J., Liang K., Hong Y. (2018). Investigating China’s Urban Air Quality Using Big Data, Information Theory, and Machine Learning. Pol. J. Environ. Stud..

[22] Berrisford L.J., Ribeiro E., Menezes R. (2022). Estimating Ambient Air Pollution Using Structural Properties of Road Networks. arXiv.

[23] Zhao Z., Wu J., Cai F., Zhang S., Wang Y.-G. (2023). A hybrid deep learning framework for air quality prediction with spatial autocorrelation during the COVID-19 pandemic. Sci. Rep..

[24] Lekinwala N.L., Bharadwaj A., Raman R.S., Bhushan M., Bali K., Dey S. (2020). Weight-of-evidence approach to identify regionally representative sites for air-quality monitoring network: Satellite data-based analysis. MethodsX.

[25] Maciejewska M., Szczurek A. Indoor Air Quality Monitoring Network Design based on Uncertainty and Mutual Information. Proceedings of the International Conference on Sensor Networks.

[26] Zaidan M.A., Dada L., Alghamdi M.A., Al-Jeelani H., Lihavainen H., Hyvärinen A., Hussein T. (2019). Mutual Information Input Selector and Probabilistic Machine Learning Utilisation for Air Pollution Proxies. Appl. Sci..

[27] Rodríguez-García M.I., González-Enrique J., Moscoso-López J.A., Ruiz-Aguilar J.J., Turias I.J. (2022). Air pollution relevance analysis in the bay of Algeciras (Spain). Int. J. Environ. Sci. Technol..

[28] Su Y., Liu Y., Li W., Xiao X., Chen C., Lu H., Yuan Z., Tai H., Jiang Y., Zou J. (2023). Sensing–transducing coupled piezoelectric textiles for self-powered humidity detection and wearable biomonitoring. Mater. Horizons.

[29] Chen C., Jiang M., Luo X., Tai H., Jiang Y., Yang M., Xie G., Su Y. (2022). Ni-Co-P hollow nanobricks enabled humidity sensor for respiratory analysis and human-machine interfacing. Sensors Actuators B Chem..

[30] Sensirion (2023). Smart Sensor Solutions. https://sensirion.com/search/news.

[31] Shrifan N.H., Akbar M.F., Isa N.A.M. (2021). An adaptive outlier removal aided k-means clustering algorithm. J. King Saud Univ. Comput. Inf. Sci..

[32] Benchekroun M., Chevallier B., Zalc V., Istrate D., Lenne D., Vera N. (2023). The Impact of Missing Data on Heart Rate Variability Features: A Comparative Study of Interpolation Methods for Ambulatory Health Monitoring. IRBM.

[33] Shannon C.E. (1948). A Mathematical Theory of Communication. Bell Syst. Tech. J..

[34] Kreer J. (1957). A question of terminology. IRE Trans. Inf. Theory.

[35] Encyclopedia W.T.F. (2023). Mutual_information. https://en.wikipedia.org/wiki/Mutual_information.

[36] Cover T.M., Thomas J.A. (2005). Elements of Information Theory.

[37] Kraskov A., Stögbauer H., Grassberger P. (2004). Estimating mutual information. Phys. Rev. E Stat. Phys. Plasmas Fluids Relat. Interdiscip. Top..

[38] Ross B.C. (2014). Mutual Information between Discrete and Continuous Data Sets. PLoS ONE.

